# Science-Based Strategies of Antiviral Coatings with Viricidal Properties for the COVID-19 Like Pandemics

**DOI:** 10.3390/ma13184041

**Published:** 2020-09-11

**Authors:** Rakesh Pemmada, Xiaoxian Zhu, Madhusmita Dash, Yubin Zhou, Seeram Ramakrishna, Xinsheng Peng, Vinoy Thomas, Sanjeev Jain, Himansu Sekhar Nanda

**Affiliations:** 1 School of Pharmacy, Guangdong Medical University, Dongguan 523808, China; s_zhuxiaoxian@163.com (X.Z.); xspeng@gdmu.edu.cn (X.P.); 2 Biomedical Engineering and Technology Laboratory, Discipline of Mechanical Engineering, PDPM-Indian Institute of Information Technology Design and Manufacturing, Jabalpur 482005, MP, India; rakeshp@iiitdmj.ac.in; 3 School of Materials and Metallurgical Engineering, Indian Institute of Technology Bhubaneswar, Arugul, Odisha 752050, India; dash.madhusmita69@gmail.com; 4 Marine Medical Research Institute of Guangdong Zhanjiang, Guangdong Zhanjiang Marine Biomedical Research Institute, Zhanjiang 524023, China; 5 Centre for Nanofibers and Nanotechnology, Department of Mechanical Engineering, National University of Singapore, Engineering Drive 3, Singapore 117587, Singapore; 6 Department of Materials Science and Engineering, University of Alabama at Birmingham, Birmingham, AL 35294, USA; vthomas@uab.edu; 7 General Administration and Technology Business Incubation Center, PDPM-Indian Institute of Information Technology Design and Manufacturing, Jabalpur 482005, MP, India; sj.director@iiitdmj.ac.in

**Keywords:** antiviral, coatings, polymeric materials, nanomaterials, metal ions and oxides, antiviral products, COVID-19

## Abstract

The worldwide, extraordinary outbreak of coronavirus pandemic (i.e., COVID-19) and other emerging viral expansions have drawn particular interest to the design and development of novel antiviral, and viricidal, agents, with a broad-spectrum of antiviral activity. The current indispensable challenge lies in the development of universal virus repudiation systems that are reusable, and capable of inactivating pathogens, thus reducing risk of infection and transmission. In this review, science-based methods, mechanisms, and procedures, which are implemented in obtaining resultant antiviral coated substrates, used in the destruction of the strains of the different viruses, are reviewed. The constituent antiviral members are classified into a few broad groups, such as polymeric materials, metal ions/metal oxides, and functional nanomaterials, based on the type of materials used at the virus contamination sites. The action mode against enveloped viruses was depicted to vindicate the antiviral mechanism. We also disclose hypothesized strategies for development of a universal and reusable virus deactivation system against the emerging COVID-19. In the surge of the current, alarming scenario of SARS-CoV-2 infections, there is a great necessity for developing highly-innovative antiviral agents to work against the viruses. We hypothesize that some of the antiviral coatings discussed here could exert an inhibitive effect on COVID-19, indicated by the results that the coatings succeeded in obtaining against other enveloped viruses. Consequently, the coatings need to be tested and authenticated, to fabricate a wide range of coated antiviral products such as masks, gowns, surgical drapes, textiles, high-touch surfaces, and other personal protective equipment, aimed at extrication from the COVID-19 pandemic.

## 1. Introduction

The presence of different microorganisms in nature may sometimes cause a detrimental impact on human health [1]. Specifically, viruses have always been regarded as increasing hazards by impairing health, as human contact with these microbes from the environment can lead to extreme illnesses and other ailments [2]. For example, tropical and subtropical countries survived an outbreak of dengue virus, known to cause the severe form of dengue hemorrhagic fever/dengue shock syndrome (DHS/DSS) [3,4]. Since the emergence of the Spanish flu outbreak (1918), influenza viral pandemics are known to appear within the interval of every 10 to 15 years [5]. Characterized by their variations in pathogenicity, the most virulent type, A influenza viruses (H1N1 and H5N1) (2009), are known to cause serious human pandemics via common transmission from animals to humans and vice versa [6,7]. Lethal varieties of coronavirus, such as severe acute respiratory syndrome-related coronavirus (SARSr-CoV) and middle east respiratory syndrome-related coronavirus (MERS-CoV) are known to cause SARS (2003) and MERS (2014) outbreaks, respectively. These coronavirus related infections were reported in several countries of North America, South America, Europe, and Asia [8,9]. Recently, an Ebola hemorrhagic fever (EHF) (2014) outbreak severely affected the living species of Africa [10,11]. In late December 2019, the emergence of a novel pneumonia drew animated attention around the world. Visualizing the chronological order, the ingenious agent that was responsible for causing the novel pneumonia has been identified as a novel coronavirus (nCoV or SARS-CoV-2) [12,13]. The outbreak of a novel coronavirus disease (COVID-19) has created a devastating challenge to the human health of various sections in the world [14]. It has caused negative social effects and massive economic damage, on a global scale. Coronaviruses contain spherical even-shaped virions, a type of enveloped RNA virus initially causing respiratory unevenness, and further leading to extreme flu [15,16]. An increased concern has arisen in the recent past with respect to a growing number of new, more virulent and pathologic viruses, such as those associated with SARS and, more recently COVID-19 [17,18].

Microorganisms constitute both bacteria and viruses [19]. Bacterial cells and viruses primarily differ from each other in terms of their size and mode of infection. More importantly, viruses reproduce by infecting a host cell and then multiplying in great numbers, causing serious illness, while the bacteria usually restricts its growth in a localized area, causing a local infection by creating an impact on a specific part of the human body [20,21]. Thus, the bacterial infections are easier to target using novel antimicrobials than the viruses. Most of the antimicrobial coatings so far developed and commercialized are antibacterial, but there are very few reports on commercialized antiviral coatings. Hence, it is highly desirable to search for potential antiviral and viricidal elements (materials and coatings) to design personal protective equipment (PPE), hygienic implements, and other devices to fight against the rise of viral pandemics and virus-associated fatal risks [22]. This review visualizes the techniques and methods that are involved in the design and development of different antiviral coatings, aiming to inspire strategies for development of coatings that are supposed to enhance antiviral efficiency, eliciting their potential application in the inhibition of COVID 19 like pandemics.

Members of antiviral coatings have been divided into three major groups (antiviral polymers, metal ions/metal oxides, and functional nanomaterials), based on the type of materials used at the contaminated sites. The methods for the treatment of virus affected substrates for preventing the virus deposition over the surfaces, using antiviral and viricidal coatings are discussed. The potential antiviral and viricidal coating technologies implemented, for design and development of a wide range of commercialized antiviral products, such as personal protective equipment (PPE), medical instrument, appliances, and hygienic implements, are discussed. Antiviral products are designed with the concept of modifying the surface, with any of the antiviral and viricidal coating compositions, using the most promising surface modification technologies [23,24,25,26,27]. Both antiviral and viricidal compositions and surface modification technologies play a major role in the destruction of viruses, by providing a thin film over the surface to retain its antiviral activity. The current review also highlights some formulations and applications of antiviral products on the basis of their antiviral compositions and activities.

## 2. Coatings Empowering Antiviral and Viricidal Properties

**Figure 1:**
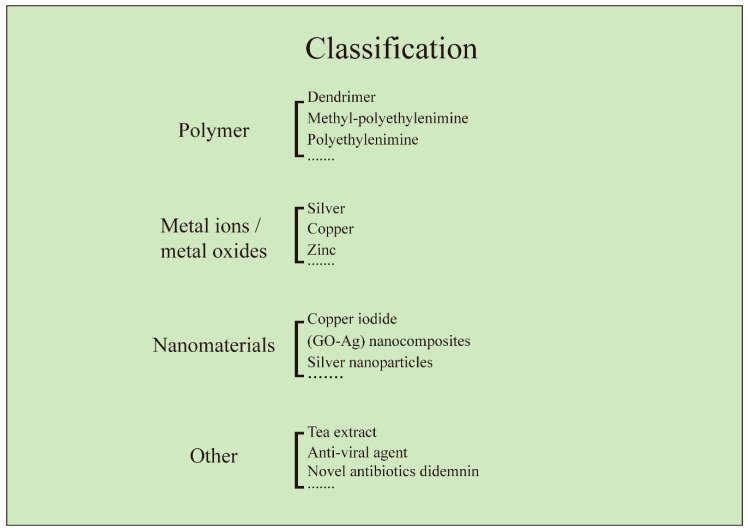
Classifications of various antiviral coating materials for the development of antiviral products.

Many assumptions and research efforts are currently being administered towards the development of vaccines against emerging viral pandemics, e.g., COVID-19 [28,29]. However, their release being uncertain, due to a unspecified timeline, attention is much needed to think about the development of antiviral surfaces, in line with sanitizing equipment and technologies. Various engineered products are analyzed, with their applications in substrate modification with antiviral polymers, antiviral metal ions/metal oxides, involving metals such as Cu, Ag, and Au, and an emerging antiviral nanomaterial [30,31,32,33,34]. The classification of all these different antivirals and viricidal coating approaches is depicted in Figure 1, and the inventions and developments under each heading are summarized in Table 1. Furthermore, some innovations in coating strategies, for the design and development of new antiviral products, have also been shared for further development of novel coating technologies against COVID-19-like pandemics.

### 2.1. Antiviral Polymers

**Figure 2:**
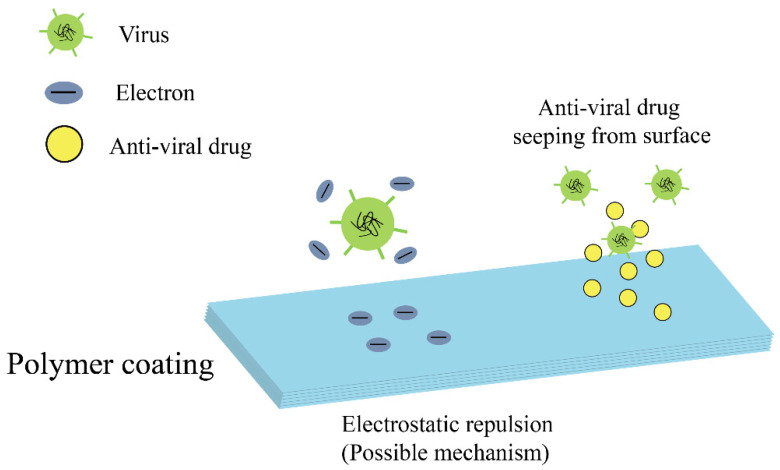
Schematic demonstration of the antiviral mechanism of polymer coatings.

A polymer is a chemical compound with the molecules bonded together in long and repeating chains. Polymers present unique properties, such as impact resistance, ductility, and elasticity, which can be tailored for various biomedical applications. Currently, a wide range of polymers have been used for antiviral composite materials. Some antiviral agents can be encapsulated in polymers to form antiviral composites, which are released upon the specific requirement. Meanwhile, some polymers have shown excellent antiviral and antibacterial effects, owing to their resistance to the adhesion of bacteria and viruses [35]. Figure 2 illustrates the antiviral mechanism of typical polymer coatings.

Polymers with antiviral and viricidal effects are mainly used as coatings, covering the surface of a wide range of substrates. Modak et al. reported an antiviral surgery/examination glove, of which the inner coating is effective to deliver an anti-infective agent within ten minutes of exposure to a liquid. The inner coating comprises of an anti-infective agent that consists of chlorohexidine, or other pharmaceutically admissible salts of chlorhexidine, and a lubricating agent that does not significantly adsorb the anti-infective agent [36]. Yao invented an antiviral composition comprising of porous plastic materials and antiviral agents, which can be incorporated into vents and filters [37]. An invention demonstrated a method for coating a substrate material with an anti-pathogenic agent, consisting essentially of povidone-iodine (PVP-I) and nonoxynol-9 (N-9). The substrate acts as a barrier to block the infection progress of pathogens in the dry state, and further releases the anti-pathogenic agent in the wet state [38]. A meshed-fabric is an excellent physical barrier to protect human body or items from virus invasion, and has been applied in many aspects of life, while polymer is one of the materials commonly used to develop antiviral and viricidal fabrics. Melvin et al. used hydrophilic polymers containing both quaternary ammonium groups, and hydrocarbon chains, to modify a fabric substrate, through photochemical immobilization, resulting in the localized action of a surfactant capable of disrupting lipid-enveloped viruses upon substrate contact [39]. Lipid-bilayer-like amphiphilic agents such as surfactants could be of interest in this regard. A material with superior water repelling properties is preferred for antiviral coatings. The superhydrophobicity of the material coating could roll off the virus or bacteria contaminated water and prevent their adhesion onto the coated surface. In such coatings, the self-cleaning activity could also be attributed through either photocatalytic/photoinduced superhydrophilicity, by incorporating titanium oxide nano-surfaces [40]. Hill et al. invented a fabric which forms a microporous polyurethane layer by a wet coagulation method [41]. This topical application of antimicrobial agents to textile fabrics can provide a certain degree of protection against bacterial and viral growth. Agricultural products are often attacked by microorganisms during long-term transportation, eventually inducing the concern of raised cost of products. Polymeric wrappings that are highly flexible and breathable are strong and inexpensive. However, none of them are able to control the growth of microorganisms in packaged, agricultural products. Gabbay et al. found that antimicrobial and antiviral properties can be enhanced by adding a small quantity of Cu, in the form of water insoluble copper oxide particles, to a polymer, and the resultant wrap package is proffered as antimicrobial for wrapping agricultural products [42]. Matsushitha et al. developed a fiber product carrying an antiviral substance, maleic acid, which is effective against avian influenza virus, human influenza, swine influenza, and norovirus. These antiviral polymer products find their potential applications in the manufacture of antiviral masks or antiviral “fiber powder” [43]. Haldar et.al. patented a glass slide painted with the hydrophobic long-chain polycation N, N-dodecyl. Methyl-polyethylenimine (N, N-dodecyl, methy l-PEI). These coated surfaces are found to be highly lethal to waterborne influenza A viruses, including not only wild-type human and avian strains, but also their neuraminidase mutants, which are resistant to currently used anti-influenza drugs. The hydrophobic polycationic coating exhibits a 100% biocidal efficiency against influenza A virus, probably by destruction of the viral lipid envelope. These aspects predict the potential feasibility of such coatings in preventing the spread of flu and other viral pandemics [44]. Larson developed multivalent antiviral agents that are covalently attached to a polymeric chain, with superior potency against target viruses, and created an antiviral surface coating that can detoxify aqueous solutions containing various viruses on contact. The amalgamation of multiple specimens of bicyclic naphthoquinone and polymers led to a significant improvement in antiviral properties. This strategy was also employed to restore inhibition for the adamantane class of influenza inhibitors against drug-resistant strains. Furthermore, this research also details the effectiveness of two FDA-approved influenza monomeric inhibitors, coated like NN-dodecyl and methyl-polyethylenimine, on both enveloped and non-enveloped viruses [45]. Mucus acts as a biopolymer matrix that coats the wet underlying cell layer to serve as a protection against various pathogenic viruses. Lieleg et al. reported a study on the isolated porcine gastric mucin polymers, which can protect the epithelia from infection by viruses, like human papillomavirus (HPV), Merkel cell polyomavirus (MCV), and a strain of influenza A virus. It was thus proposed that purified mucins could be used in a variety of antivirals to supplement personal hygiene products [46]. Matthews et al. patented an antiviral composition comprising a dendrimer, either a polyamidoamine or polylysine dendrimer, having a plurality of terminal functional groups, particularly a sulfonic acid-containing, carboxylic acid-containing, or trimethylammonium-containing moiety. This composition is therefore well suited to prophylactic and therapeutic use, as antiviral agents in humans [47]. Tavakoli et al. proposed (PEG)-coated ZnO-nanoparticles, as an effective antiviral against herpes simplex virus type 1. The concept of PEGylation to a nanoparticle could be considered as an effective process to enhance the antiviral activity of nanoparticles, apart from the advantages of control over their cytotoxicity [48].

Currently, we come across a variety of approaches to create sterile environments, those that suspend the growth of viruses. As mentioned, the functional polymers have well demonstrated their antiviral efficacy on a wide range of enveloped viruses. Coatings of these antiviral or viricidal polymers and functional organic–inorganics on PPE and other surfaces may be considered as a viable approach to prevent the growth of COVID and other emerging viruses.

### 2.2. Antiviral Metal Ions/Metal Oxides

**Figure 3:**
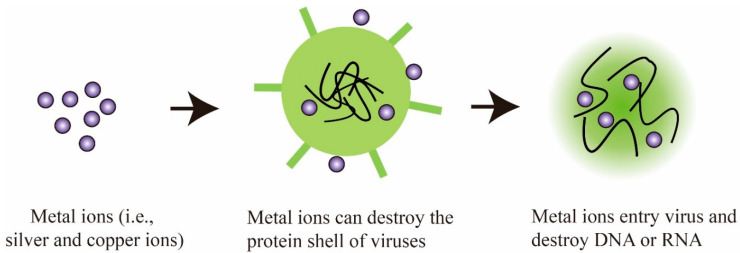
Schematic showing the antiviral mechanism of metal ions.

In the past decades, a large number of metal ions and metal oxides have been investigated for their antiviral and viricidal activity, particularly silver, copper, and zinc-based ions/oxides. In general, these metal materials show lower toxicological effects and high antiviral activity compared to other metals, making them a popular fit for this kind of application. Conventionally, both the metal ions and metal oxides exhibit similar antiviral mechanisms of control over different viral strains. For instance, metal ions can adhere to the viral envelope, the membrane of cells, and then enter the interior, destroying genetic materials such as DNA and RNA [49]. The possible mechanism of action typical of metal ions/oxides is illustrated in Figure 3.

Silver and copper ions present the broadest spectrum of antiviral action among the different metal ions [50]. The use of such materials in coatings and filters accounts for their prime area of application in the elimination of viruses. Metal ions and metal oxides are usually mixed with different materials to form composite coatings. Hodek et al. developed a hybrid coating containing silver, copper, and zinc cations, which was fabricated through radical polymerization, via a sol-gel method. The developed hybrid coatings were able to show viricidal activity against HIV and other enveloped viruses, including dengue and herpes simplex [51]. In addition, a large number of studies have reported coating materials containing metal ions (i.e., silver, copper, zinc), which have demonstrated an excellent antiviral ability with long-term, persistent effects [30,31,32,33,51,52,53,54,55]. Smaller metal nanoparticles (NPs) have the intrinsic ability to pass through the cell membrane and hinder post-attachment virus replication [56]. More recently, it was reported that a solution containing a metal oxide (TiO_2_) and metal ion (Ag+) was used for street disinfection in Milan, Italy. Ueda et al. patented a copper complexed TiO_2_ dispersion liquid and a coating agent composition, with a binder resin that can form a transparent coating film having an increased antiviral performance [57]. Charan et al. demonstrated the antiviral activity of arsenic oxide (As_2_O_3_) and antimony oxide (Sb_2_O_3_) against bacteriophage virus. The hydroxyl radical oxidation mechanism promotes the diffusion of the heavy metal oxide disinfectants into the cell membrane, and thus leading to the disruption of the cell [58]. Mucus from infected people is the prime source of a very high concentration of viruses and is aerosolized by a sneeze and cough. Kelly et al. patented an antiviral tissue paper, composed of one or more fibrous plies and an antiviral composition. The antiviral composition comprises of a viricidal, effective water-soluble metal ion, aluminum, copper, and mixtures. This developed tissue paper not only showed an excellent antiviral effect, but also reduced skin irritation due to its mildness to skin. When virus-containing mucus was added to the tissue paper, the water-soluble metal ions killed certain strains of viruses on contact with the tissue [59]. Cleaning with bleaching agents is highly desirable to maintain better sanitary conditions. These methods can effectively create a virus free surface by cleaning, but it has a high chance of relapse of viruses on the same surface substrates [60,61]. Furthermore, the bleaching agents can affect the surfaces on their use for a long time. Metal ions and oxides can be mixed with other antiviral materials to achieve viricidal activity and reduce the deleterious impact to the surface. Gabbay et al. added copper ion, in powder form, to polymeric materials, which were used to form a condom sheath, surgical tubing, or surgical gloves [62]. Metal ions and metal oxides can also be used as an antiviral coating for the treatment of localized infections. For example, an antiviral composition was invented containing a thiosulfate salt and at least one of the thiosulfate complex salts of a metal (silver, copper, and zinc) and a porous particulate carrier. This composition can be applied in medical devices and appliances, and as antiviral ointments by loading the composition into the ointment base [63]. Trogolo et al. reported an antiviral composition with combinations of silver and copper ion sources, or a single source of either, which were found to be effective, and claimed as a possible application for the treatment of the diseases incidental with SARS [64].

Broad-spectrum therapeutic effects promise to be an appealing use of metal ions/metal oxides in virus-targeting formulations. The use of most of these antiviral metal ions and oxides, in combination with functional polymers on the surfaces and PPEs, could be considered as an attractive strategy to deactivate the novel coronavirus strain and its spreading. Moreover, the impregnating/coating of classical antibacterial metals/metal oxides in nano/micro size scales, such as copper oxide, zinc oxides, titanium oxide, silver oxide etc. onto disposable N95 respiratory mask layers, showed that the mask layers had an anti-viral activity against human influenza A virus (H1N1) and avian influenza virus (H9N2) [65,66]. These metals and metallic oxides could be useful in integrating anti-viral activity onto the surfaces of masks and other PPE to reduce the risk of infection and environmental contamination, with novel coronavirus, for COVID-19.

### 2.3. Antiviral Functional Nanomaterials

**Figure 4:**
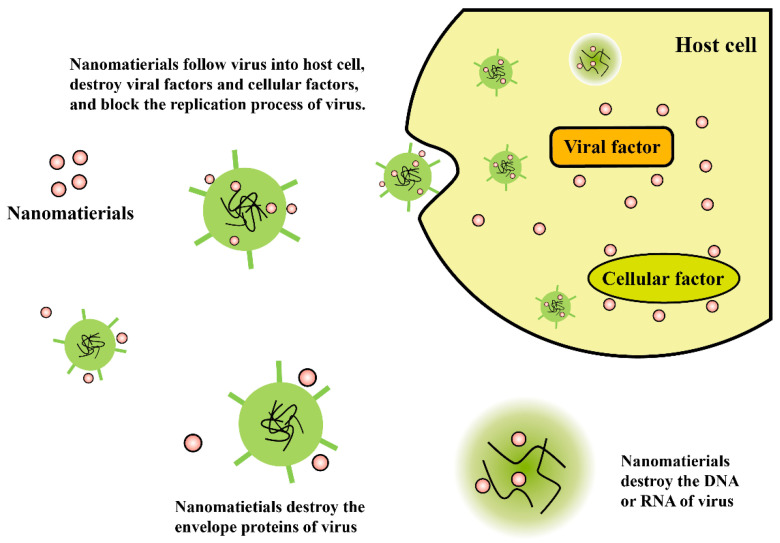
Illustration of antiviral mechanisms of functional nanomaterials.

According to the nanoscale properties of viruses, it’s possible to develop certain hybrid nanomaterials with multiple functionalities, to achieve viricidal effects, since they are considered to be highly effective in controlling viral infections. Figure 4 illustrates the antiviral mechanisms of functional nanomaterials. The absolute biological effect could be determined by the physical and chemical characterization, and by functionalized chemical modification of the materials. In this section, we have furnished specific examples of selected studies that demonstrate practical applications and working mechanisms of multiple nanomaterials, in relation to inhibition of viruses or virus infections.

A study shown by Park, et al. demonstrated the use of polyurethane, Quat-12-PU, nanomaterials as an antiviral reagent [67,68]. Quat-12-PU could be processed into solutions, nanoparticles, and nanofibers, exhibiting strong antiviral activities when coated onto a surface or processed into electro-spun nanofibers. The surface of Quat-12-PU was able to inactivate the enveloped virus (e.g., influenza viruses), but not the non-enveloped ones (e.g., poliovirus) [67]. The incorporation of Ag+ ions in several studies shows a greater capacity for destruction of virus. On the other hand, the performance of the antiviral nanomaterials could be enhanced with the combination of metal oxides and cationic surfactants [69]. Nano-sized copper (I), iodide (CuI), silver (Ag), and gold (Au) particles showed broad spectrum antiviral activity by generating free radicals and ionic charges [30,70,71,72]. Several studies demonstrated the reduction of virus titer in a dose-dependent manner upon incubation with these nanoparticles [73]. Hang et al. reported the antiviral efficacy of cuprous oxide nanoparticles (CO-NP’s) against hepatitis C virus in a HCVcc/Huh7.5.1 culture system. The HCV particles are bound to hepatic cells, containing CO-NP’s acting as an anti-HCV agent, by controlling the ingress of viral strains into the cells [74]. Fujimori et al. investigated the antiviral activity of nanosized copper iodide (CuI) particles, with an average size of 160 nm, and found that Cu^+^ in aqueous solution might behave as a catalyst in a Fenton-like reaction, and exerted an antiviral activity through generating hydroxyl radicals, leading to the degradation of viral proteins (such as hemagglutinin and neuraminidase) [75]. This antiviral property would enable CuI nanoparticles to be applied to filters, masks, or protective clothing, by blending with, or coating on, polymer base materials [31]. Metal nanoparticles, such as Ag and Au (of sizes below 100 nm), showed antiviral and viricidal activity against a broad spectrum of viruses, reducing the viral infectivity [30,32,33,34,76]. In the case of metal nanoparticles, a direct interaction between nanoparticles and virus surface proteins could be observed, as demonstrated in Figure 3. Ag nanoparticles have mainly been studied for their antimicrobial potential against bacteria, but PVP coated Ag nanoparticles (of size 69 ± 3 nm) induced virus inactivation by inhibiting the surface attachment of respiratory syncytial virus [51]. Ghosh et al. developed NANOVA HYGIENE + TM omniphobic antimicrobial coating, containing Ag NP’s as bioactive particles, to stop the replication of spike glycoprotein and virus nucleotides. The coating was hypothesized as viricidal against poliovirus and non-enveloped RNA viruses [77]. Similarly, sialic acid, functionalized with Au nanoparticles in the range of 14 nm, could inhibit the attachment of influenza viruses to a substrate [32,34]. Lysenko et al. embodied a couple of specimens with Au NP’s, with one covered with a SiO_2_ shell as a core shell structure, and the other as an Au-SiO_2_ carrier nanoparticle system, to use them against adenoviruses [78]. Mori et al. reported a similar study on Ag nanoparticles and chitosan composites. The Ag nanoparticles could maintain antiviral activities by inhibition of the respiratory enzymes, and thereby controlling the binding between virus and host cell. The concentration of the Ag nanoparticles in the nanoparticle/chitosan composites was directly proportional to the antiviral activity of the composites [79].

Functional nanoparticles have a high surface area to volume ratio. Moreover, these active materials, in nano size, have a similar physical size to that of viruses. The available reports described, should evolve and inspire an extensive level of knowledge about the unexplored antiviral properties of nanomaterials, to use against emerged COVID-19. Various functional nanoparticles of metals and their oxides, graphene, and metals in combination with graphene or graphene oxides, are currently under exploration against COVID-19 [80]. Graphene oxide (GO) sheets with silver particles, i.e., (GO-Ag) nanocomposites, have been developed and could be used in personal protective equipment against enveloped and non-enveloped viruses [81]. The role of functional nanomaterials and nanotechnology is highly relevant to counter the COVID-19 “virus” nano-enemy. Nano intervention is discussed in terms of designing effective nanomaterials-driven antibacterial/viricidal coatings, to counter the limitations of conventional antiviral coatings.

## 3. Antiviral Products

Coating materials are continuously evolving, and their practical applications in antiviral products have great public health, and social, importance. Chu et al. have demonstrated, in a systematic analysis, that wearing face masks may protect people against infection by coronaviruses [92]. This report motivates and prompts the development of various materials to manufacture the antiviral products against COVID-19. CytaCoat is the first antimicrobial coating developed with biocompatible organic components to inactivate SARS-COV-2 (COVID-19). Unlike the inventions presented in previous sections, CytaCoat is not dependent on toxic metal ions or metal oxides, hence its applicability in face masks to prevent the spread of novel coronaviruses [93]. Another invention has been reported; a lightweight and disposable antiviral respirator mask, which comprises a filter that may preferably include a passive layer and an active disinfectant layer. This market available product was able to kill viruses with a small size, hardly blocked by only passive filters [83]. In another embodiment, an invention used a cartridge or filter inserted in a gas or respirator mask for destroying hazardous viral substances [94]. In the absence of vaccination for the emerging viral pandemics, respirators and masks can be worn to prevent transmission of airborne pathogenic aerosols and control the diseases such as influenza and the coronaviruses responsible for COVID-19 [95]. These devices are capable of trapping the viruses floating in the ambient air at a higher rate, followed by inactivating the viruses trapped from the air. Lightweight and breathable fiber fabrics containing antiviral and viricidal properties can be developed to replace the intermediate layers of currently available masks, to use them as personal protective clothing, with necessary antiviral and viricidal activity. These potential fabrics can be developed using antiviral polymers and a mixture of antiviral or viricidal nanoparticles and polymers. The special grade composite polymer solutions can either be coated onto the surface or can be electro-spun into the fiber fabrics to address the antiviral or viricidal activity [66,96,97].

The fluidic compositions are developed as supportive coatings with highly efficient antiviral properties. The fluid compositions in particular are premeditated to cover the articles that are plausible to carry the viruses, especially toys, health devices, notes, cards, etc. The presence of virus on the subjects may lead to contamination, while the incorporation of fluidic compositions may prevent these events. Rossett et al. proposed a fluidic composition that comprises of at least one viricide, like lauric acid and essential oils like laurel essential oil, or soya bean oil, having an antiviral activity. The ability of the viricide in the composition makes the surface look transparent, with an effective coating [86]. Fox et al. proposed a potential antiviral composition that includes a powdered substrate solution and an organic acid. This solid combination could be applied directly onto the substrate to destroy rhino and rota viruses. The composition plays a major role in the destruction of non-enveloped and enveloped influenza viruses, and also provides a thin film of organic acid on the surface to continue its antiviral activity. These antiviral compositions present some strong efficacious solutions, against a broad-spectrum of viruses, and also makes it feasible to produce at a lower cost [98].

The current limitation faced by the antiviral coatings is their usage in wet areas like sinks and faucets, due to their resistance to water. To resolve these issues, Watanabe et al. combined a particular polyhydric carboxylic acid with quaternary ammonium salt, which serves as an antiviral component used in coating material like paints and varnishes, to form highly durable antiviral, water-resistant films [83]. Torkelson et al. investigated a quaternary ammonium silane compound attached to sand filters to effectively treat bacteria and virus from drinking water. This could destroy certain strains of *E.coli*, Ms2 colliphage, poliovirus type3, and Adenovirus type2, thus demonstrating its application in both antimicrobial and antiviral water filters [99]. These antiviral compositions provide the advantages of a broad-spectrum of viral control. Continuous antiviral activity is not only maintained due to the presence of these compositions, but also this activity is sustained because a residual barrier film of composition ingredients is incorporated onto the objects/surfaces.

## 4. Conclusions and Future Perspectives

In this review, specific coating materials that inactivate viruses have been discussed. Various strategies involved in the development of antiviral and viricidal coatings, like modifying the surface of a substrate via antiviral polymers, incorporation of metal ions/oxides, and functional nanoparticles were discussed. The antiviral efficacies of the developed coatings were detailed, and their possible and promising applications were further correlated with emerging viral pandemics, like COVID-19. There are a few areas that may need special attention to improve the existing technologies to fight against the current pandemic. 1. Processing of nano-sized metal particles and focusing their utility either in their original form or mixing them with polymers, to prepare novel functional coatings. 2. Chemical modification of the polymers to prepare a highly effective antiviral formulation. 3. The functional modification of the antiviral or viricidal nanoparticles, using other chemical moieties for better integration within a coating composition. In addition, immaculate procedures have to be identified in processing the antiviral coating materials. Based on recent progress, both inorganic polymer-based, and nanostructured, coating materials mentioned in this review have demonstrated the properties of superhydrophobicity, photo-induced superhydrophilicity, and excellent surface topography of the coated surfaces. An important problem that needs considerable attention is the long-term persistence of the virus particles on the surface layer of face masks, putting them at higher risk level during their usage and disposal. Hence, the manufacturing of optimized face masks by the application of a metal ions, consisting of nanoparticles on the surface of the filtering layer, could be considered as a viable approach for instantaneous elimination of viruses. Moreover, self-cleaning coatings on the filtering layer of the masks, could be applied to avoid the attachment of infectious microdroplets on face masks. The current coronavirus responsible for COVID-19 is transmitted not only through droplets, but also via various surfaces that can convey the virus from one person to another. Furthermore, research shows that the virus remains viable on various surfaces for extended periods of time, for days and even longer. Therefore, there is a clear need for durable anti-viral coatings that can be sprayed or painted on surfaces, just like paint or varnish, and that will prevent viral transmission. Finally, this review attempts to summarize and improvise the extension of present techniques, with certain modifications, for the prevention of COVID-19, and further inspire future antiviral strategies. Apart from viable functional coatings, conductive nanocoating (photo-thermally or electrically) of materials/metals onto surfaces, via robust and sterile methods, such as non-thermal plasma process, could be used in the future, not only for sterilizing surfaces, but to deliver nanomaterials-driven anti-infective surfaces for prolonged/reusable applications.

## Figures and Tables

**Table 1:** Coatings used for antiviral and viricidal applications. NA: Not applicable.

Type of Coating	Coating Materials	Mechanism	Effectiveness Conditions	Average Time Duration to Destroy Viruses	References
Polymer	Polymers containing both quaternary ammonium groups and hydrocarbon chains	Creating localized surfactancy	Inactivation of lipid-enveloped viruses	−NA−	[39]
Polymer	Microbicidal polycation N,N-dodecyl, methyl-polyethylenimine	−NA−	Destruction of human bacteria pathogens (Staphylococcus aureus and Escherichia coli) and two common strains of influenza virus	Up to 5 days	[23]
Polymer	An anti-infective agent selected from the group consisting of chlorhexidine and pharmaceutically acceptable salts of chlorhexidine	−NA− Blocking adsorption sites of anti-infective agent	−NA−	−NA−	[36]
Polymer	Methyl-polyethylenimine (N,N-dodecyl,methyl-PEI)	−NA−	Highly lethal to waterborne Influenza A viruses, including wild-type human and avian strains	Up to 5 days	[44]
Polymer	Polyethylene (preferred polyolefin)	−NA−	Porous plastic materials with antiviral agent	2−3 days	[37]
Polymer	Polyethylenimine	−NA−	Antiviral surface coating that can detoxify aqueous solutions containing various viruses	−NA−	[45]
Polymer	PVP-I and N-9	−NA−	Dual or multilayered format antiviral coating that imparts antipathogenic properties to substrate	−NA−	[38]
Polymer	A dendrimer such as a polyamidoamine or polyly sine having a plurality of terminal groups	Attachment of Ionic moieties	Antiviral activity against human immunodeficiency (HIV) and other enveloped viruses	−NA−	[47]
Polymer	Porcine gastric mucin polymers	Shielding effect	Protect an underlying cell layer from infection by small viruses like human papillomavirus (HPV), Merkel cell polyomavirus (MCV), or a strain of influenza A virus	−NA−	[46]
Polymer	A polymeric component selected from the group consisting of a polyamide, a polyester, an acrylic, a polyalkylene, and mixtures thereof	Precipitation and reduction	Action of Cu^++^ particles as antiviral agents	Less than 4 h	[42]
Polymer	A polymer containing a maleic acid component as a monomer unit in a polymer chain thereof	Action of metallic particles with copolymerized fiber	Effective against an avian influenza virus	−NA−	[43]
Metal ions/metal oxides	Copper ion, silver ion or both	Ion exchange type	Action of single source of both Ag++ and Cu^++^ ions in treating virally contaminated surfaces	Less than 4 h	[64]
Metal ions/metal oxides	Cu in powder	Electrolytic Plating	Action of Cu^++^ ions	Less than 4 h	[62]
Metal ions/metal oxides	Silver, copper and zinc	Washing with PBS	Action of Ag++, Zn++ and Cu^++^ ions showed viricidal activity against (HIV-1), and other enveloped viruses	Less than 4 h	[51]
Metal ions/metal oxides		Reduction/oxidation reaction on surfaces of photocatalytic particles	Inactivating Influenza viruses and Norovirus	Less than 4 h	[52]
Metal ions/metal oxides	A copper complex titanium oxide dispersion liquid	Dispersion	−NA−	Less than 4 h	[57]
Metal ions/metal oxides	Cuprous oxide particle dispersion liquid and a binder resin	Coupling effect	−NA−	Less than 4 h	[82]
Metal ions/metal oxides	Water soluble metal ions include aluminum, copper, and mixtures thereof	Hydroxide formation	Water soluble metal ion has the ability to kill certain strains of viruses	4−8 h	[59]
Metal ions/metal oxides	An antiviral composition consisting of a thiosulfate complex salt coated with a material layer like Silicon dioxide of a metal like silver, copper and zinc	Releasing salts and by attaching chemotherapeutic agents to complex	The composition releases its salts into the contaminated sites	−NA−	[63]
Metal ions/metal oxides	Metal oxides or metalloid oxides, such as, e.g., TiO_2_, ZrO_2_, SnO_2_, ZnO, and SiO_2_	oligodynamic effect	−NA−	4−8 h	[69]
Metal ions/metal oxides	Antiviral activity of Arsenic oxide (As_2_O_3_) and Antimony Oxide (Sb_2_O_3_)	Hydroxyl radical oxidation, diffusion of disinfectant	Excellent viricidal property on viral strain bacteriophage	−NA−	[58]
Nanomaterials	Nanosized copper (I) iodide (CuI)	Hydroxyl radical formation	CuI particles showed antiviral activity against influenza A virus of swine origin	Less than 4 h	[31]
Nanomaterials	Silver nanoparticles	Blocking interaction	inactivating many viral strains from lipid envelope viruses	Less than 4 h	[73,79]
Nanomaterials	(GO-Ag) Nanocomposites	Washing	Antiviral activity of nanomaterials on non-enveloped viruses	Less Than 4 h	[81]
Other	A quaternary ammonium salt and a polyhydric carboxylic acid having a C6 or more hydrocarbon group and two or more carboxyl groups	Preventing elution of salts in water thus maintaining antiviral effect	Water resistant antiviral coating	2−3 days	[83]
Other	An antiviral agent contains a powder obtained by baking (calcining) dolomite and hydrating a part thereof	Filtration	An antiviral coating effective for Coronavirus	−NA−	[84]
Other	An antiviral agent includes an inorganic solid acid having an acid site concentration of more than 0.005 mmol/g	Dispersion	Excellent in heat resistance and maintains the inactivating effect on viruses	−NA−	[85]
Other	The fluid compositions consist of at least one viricide like lauric acid and essential oils like laurel essential oil, soybean oil	−NA−	−NA−	2−3 days	[86]
Other	A therapeutically effective amount of an essential oil	Presence of different functional groups in essential oils.	This method prevents a respiratory infection in a mammal	−NA−	[87]
Other	Novel antibiotics didemnin A, B, C, D, and E (didemnins)	Drug delivery system (DDS)	Inactivate variety of DNA and RNA viruses.	−NA−	[88]
Other	Antiviral Filter air Cleaner impregnated with tea extract	Filtration	Disable influenza virus	−NA−	[89]
Other	Tea extract, other herbals, and phytochemicals like Curcumin	Filtration	Acting against Influenza virus, hepatitis C Virus, HIV	−NA−	[90]
Other	Chitosan incorporated neem seed extract (*Azadirachta indica*)		Higher antibacterial activity against gram-positive and gram-negative bacteria	−NA−	[91]
